# Variation in Human Bone Collagen Turnover Among Skeletal Elements

**DOI:** 10.1002/ajpa.70199

**Published:** 2026-01-21

**Authors:** Olivia Hall, Shari L. Forbes, Paul Szpak

**Affiliations:** ^1^ Department of Anthropology Trent University Peterborough Ontario Canada; ^2^ Department of Chemistry and Biochemistry University of Windsor Windsor Ontario Canada

**Keywords:** ^14^C, bomb carbon dating, dietary analysis, isotope analysis, turnover rate

## Abstract

**Objective:**

Understanding tissue turnover rate is crucial for isotopic analysis. The stable isotope composition of collagen is often studied in archeology and paleontology, yet bone collagen turnover rates across various skeletal elements remain underexplored. The turnover rate of various skeletal elements in humans was explored to improve the accuracy and reliability of stable isotopic studies.

**Materials and Methods:**

Bone collagen turnover rates were quantified in multiple human skeletal elements using the bomb carbon dating method. Fifty‐three skeletal elements from seven donors, aged 54–78, from a human decomposition facility in Québec, Canada, were sampled.

**Results:**

Turnover rates varied significantly among skeletal elements, ranked from slowest to fastest as follows: ulna, humerus, femur, rib, pelvis, and vertebra. Turnover rates must markedly decline through life, and the isotopic composition of bone collagen samples therefore represents a generally long term (years to decade) but uneven (heavily weighted toward earlier life for elements with slow turnover rates) picture of the life history.

**Discussion:**

There can be significant variation in turnover rates within skeletal elements, which researchers should account for in isotopic studies. For stable isotope studies aiming to reconstruct temporal variation in life history, the rib and femur may be among the least suitable paired elements for sampling. Such studies should instead select vertebrae and ulnae due to their highly distinct turnover rates.

## 1 Introduction

**FIGURE 1:**
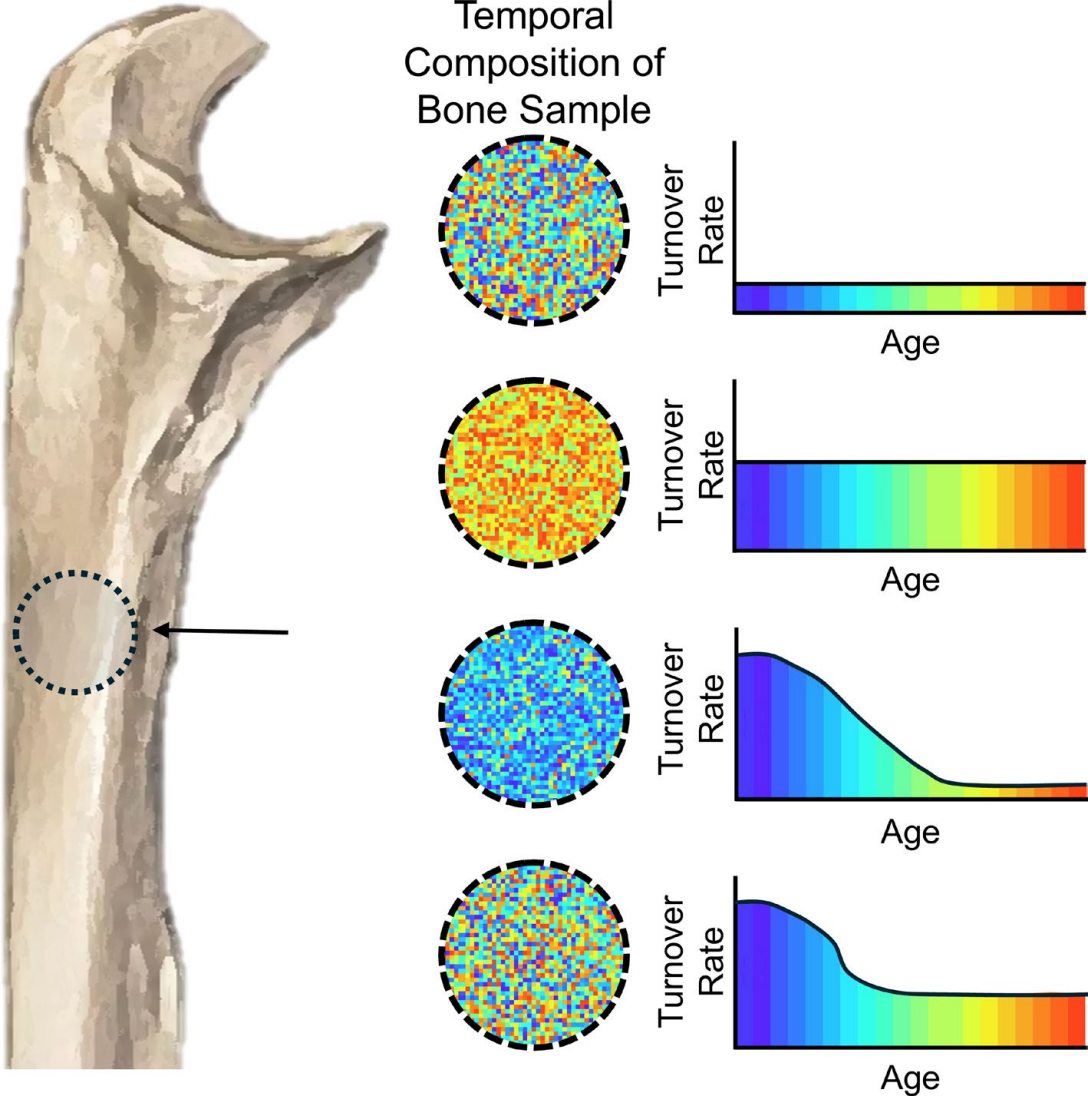
Schematic showing four possible models of bone turnover for the sampled region of the ulna (circled) and their implications for the temporal composition of that sample.

In archeology, skeletal remains act as time capsules, offering insights into ancient human populations. Bones can reveal information about environments, societal differences, specialized roles, migration, and more (Makarewicz and Sealy 2015). Bone collagen is of particular importance in archeology as it can be preserved over tens of thousands of years (e.g., Rey‐Iglesia et al. 2019; Peters et al. 2023), providing an easy‐to‐isolate and relatively pure analytical substrate for stable isotope analysis. Bone collagen undergoes continuous remodeling throughout the life of an individual (Safadi et al. 2009). The speed at which bone remodels is referred to as the turnover rate, expressed as the average proportion that has remodeled annually. As stable isotope scientists rely on collagen to deduce crucial life history details (Makarewicz and Sealy 2015; Reitsma 2013), understanding the temporal dimension of this protein is pivotal (Figure 1), but remains critically understudied. The turnover interval is the period of life represented by a sample of bone collagen, as reflected by the four circles in Figure 1.

The most important underpinnings of stable isotope studies are the ability to accurately and precisely measure the isotopic compositions, knowing the relationship between the isotopic compositions of sources (i.e., foods) and mixtures (i.e., consumers), and understanding the rate of tissue formation or turnover. For bone collagen, there are well‐established methods for accurately and precisely measuring its isotopic composition, taking into account alteration in the burial environment and contamination. Furthermore, many studies have quantified trophic discrimination factors for a range of taxa and addressed issues of macronutrient routing (Ambrose 2000; Ambrose and Norr 1993; Jim et al. 2004). Comparatively, the uncertainty surrounding bone collagen turnover rates remains a major obstacle to producing accurate reconstructions of ancient human diets. Few studies have provided quantitative (i.e., producing annual turnover rates) or even qualitative (i.e., ordering relative turnover rates of elements from slow to fast) turnover rates for human skeletal elements (e.g., Bryant and Loutit 1963; Fahy et al. 2017). Hedges et al.'s (2007) study of femoral cortical collagen turnover using bomb curve dating concluded that the femur's turnover interval exceeds 10 years and can reach up to 30 years in older individuals. Despite this, their findings are often misrepresented, applied uncritically, or extrapolated to other skeletal elements. For example, Gregoricka and Ullinger (2022) state that “For adults, [stable isotope] values derived from bones such as ribs approximate an average of the last 10 years of life, while slower turnover rates of up to 30 years have been reported for the thicker cortical bone of femora (Hedges et al. 2007)”. Clearly, the data from Hedges et al. (2007) cannot speak to the turnover rate of the rib, and while 30 years could be an accurate upper limit for the femur, this is not a value supported by Hedges et al. (2007). Moreover, such estimates are likely age‐dependent, and assuming a discrete turnover period fails to account for the persistence of adolescent collagen within adult bone. Similar misinterpretations appear in numerous studies employing bone collagen as an analyte and citing Hedges et al. (2007) to justify turnover rate assumptions, highlighting persistent uncertainty regarding the true temporal resolution of bone collagen samples.

There is little experimental evidence regarding the turnover rates for different skeletal elements. Many have referenced the work of Cox and Sealy (1997), which suggested that paired comparisons of elements with slow (more cortical bone) and fast (more trabecular bone) elements could be used to detect life history changes. Commonly, such comparisons have involved the femur and the rib (Lamb et al. 2014; Gregoricka and Ullinger 2022; Pollard et al. 2012; Schroeder et al. 2009; Drtikolova Kaupova et al. 2021), but some studies have found that these elements are not well‐suited for such comparisons because of more comparable turnover rates (Fahy et al. 2017; Quinn 2024). While the potential of nondental samples for generating some temporally resolved life history information is large, it relies on well‐characterized turnover rates for different skeletal elements. Utilizing new AMS ^14^C measurements of purified bone collagen of human donors from a Canadian human decomposition facility, this paper aims to address this critical knowledge gap by assessing turnover rates across the human skeleton to more accurately characterize the percentage of bone attributable to any specific year of life.

### 1.1 Bomb Pulse Radiocarbon Dating

**FIGURE 2:**
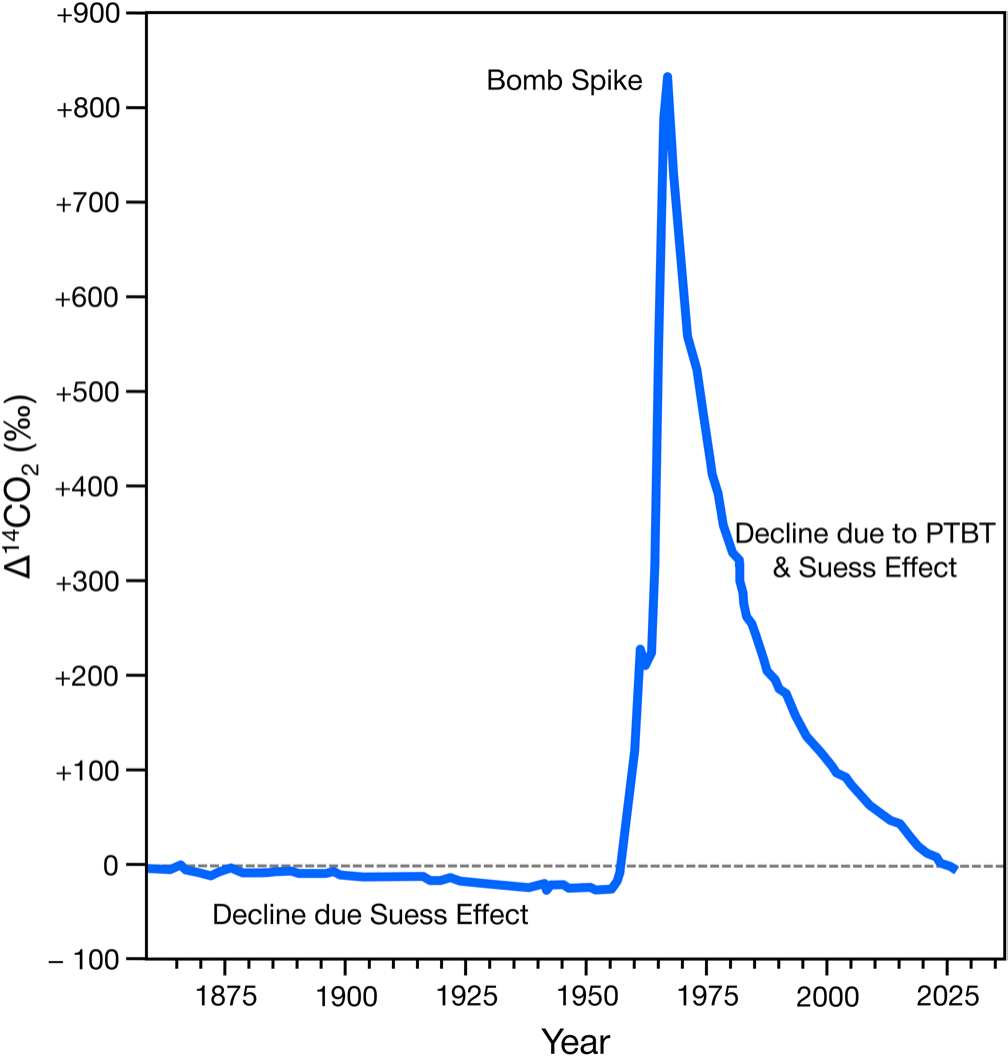
Northern hemisphere atmospheric ^14^CO_2_ levels, highlighting the decline in ^14^C due to the burning of fossil fuels beginning in the late 19th century (Suess Effect), the peak in ^14^C due to thermonuclear weapons testing (Bomb Spike) in 1963, and the decline in ^14^C due to the Partial Nuclear Test Ban Treaty (PTBT) and continued Suess Effect. Data used to produce this graph are from Graven et al. (2017), and Hua et al. (2022), summarized in Table S1.

Since the late 1800s, increased fossil fuel production has released large amounts of CO_2_ depleted in ^14^C into the atmosphere, creating a long‐term decline in atmospheric ^14^C levels, often referred to as the Suess effect (Figure 2; Baxter and Walton 1970; Taylor 2016; Suess 1955). Atmospheric ^14^C levels spiked in the mid‐1950s due to thermonuclear weapons testing (a process that produces ^14^C in a manner analogous to natural cosmic radiation), which nearly doubled ^14^C in terrestrial organics from 1955 to 1963 (Figure 2) (Burr 2015). After the 1963 agreement to end above‐ground nuclear testing (PTBT), ^14^C levels began declining, driven primarily by the accelerated burning of ^14^C‐depleted fossil fuels, a trend that continues today (Figure 2). Since the historic ^14^C levels for each year are well documented through tree rings and other records, radiocarbon analyses of organic materials from the decline period have been used to address various questions, such as determining the age at death in forensic cases (Calcagnile et al. 2013; Kutschera 2022; Ubelaker et al. 2022; Wild et al. 2000), ecological conservation issues (Andrews et al. 2013; Cerling et al. 2016; Kutschera 2022), and bone turnover rates (Hedges et al. 2007; Quinn 2023; Quinn 2024). This method is well‐suited for quantifying bone turnover as the rapidly changing atmospheric ^14^C values would be incorporated into plant and animal tissues formed in a given year and subsequently synthesized into bone collagen shortly thereafter. This method works best for individuals that were born close to the spike (e.g., an individual born in 1955 that died in 2020) and is more complicated for individuals that lived significant periods of their lives on either side of the spike (e.g., an individual born in 1920 that died in 1985) since there are 2 years (on either side of the spike) with the same atmospheric ^14^C content.

## 2 Materials and Methods

### 2.1 Materials

Samples for this research were acquired from the Research in Experimental and Social Thanatology/*Recherche en Sciences Thanatologiques [Experimentales et Sociales]* facility, also referred to as REST[ES], which is a facility associated with the Université du Québec à Trois‐Rivières (UQTR) near Becancour, Québec, Canada. REST[ES] is a high‐security outdoor facility that holds the remains of individuals who have donated their bodies to UQTR for forensic science research purposes. Human ethics approval for sample collection from the donors was approved by UQTR with certification numbers CER‐19‐261‐07.10 and SCELERA 22–06. The research completed here is considered to be secondary use of the approved samples and is consistent with the existing research agreement, which was ratified by Trent University. The donors provided detailed information about their life prior to death (Table 1), and the facility is able to anonymously use this information for research and to understand physiological and demographic differences between the individuals.

**TABLE 1:** Donor information for the individuals sampled for this research from the REST[ES] facility in Quebec, Canada.

Donor #	Health details	Birth year	Death year	Sex
3	Parkinsonism syndrome	1951	2020	F
5	Metastatic melanoma	1948	2020	M
6	Metastatic melanoma	1944	2021	M
10	Lung neoplasia	1943	2021	M
11	Metastatic lung cancer	1967	2021	M
12	Lung neoplasia, on Alendronate for Osteoporosis	1948	2021	F
17	Paralysis	1949	2022	M

Donors were selected based on their state of decomposition to ensure other tissues remained unaltered for use in various other research projects; thus, only individuals with accessible skeletal material were sampled. The choice of skeletal elements depended on accessibility, so equal numbers of each element could not be obtained across donors. However, an attempt was made to take samples from roughly the same position across elements, although because of the partially decayed state of the remains, this was not always possible. A variety of skeletal elements were collected from each donor (see Table 2), with ~100 mg sections removed from each bone for further processing. In some cases, sections were subdivided to assess intrabone variability. For one sample (20506), two ~100 mg sections were taken from the same mandible to explore potential turnover rate differences within the same skeletal element. Another rib sample (20526) was subdivided to sample both cortical (A) and trabecular (B) regions from the same rib.

**TABLE 2:** Overview of the samples used in this study.

Donor number	Lab ID number	Skeletal element	Side/sample specifications
3	20491	Femur	Right, midshaft
3	20492	Rib	Left, midshaft
3	20493	Vertebra	Thoracic, body
3	20494	Ulna	Right, midshaft
3	20495	Public symphysis	
3	20496	Iliac crest	
3	20497	Humerus	Right, midshaft
5	20498	Femur	Right, midshaft
5	20499	Rib	Right, midshaft
5	20500	Ulna	Right, midshaft
5	20501	Pubic symphysis	
5	20502	Vertebra	Thoracic, body
5	20503	Iliac crest	
5	20504	Humerus	Left, midshaft
6	20505 A1	Femur	Right, midshaft
6	20505 A2	Femur	Right, midshaft
6	20505 B1	Femur	Right, midshaft
6	20505 B2	Femur	Right, midshaft
6	20505 C1	Femur	Right, midshaft
6	20505 C2	Femur	Right, midshaft
6	20505 D1	Femur	Right, midshaft
6	20505 D2	Femur	Right, midshaft
6	20506 A	Mandible	Bases of the body of right side of mandible, taken roughly center
6	20506 B	Mandible	Bases of the body of right side of mandible, taken roughly center
6	20507	Ulna	Left, midshaft
6	20508	Rib	Left, midshaft
6	20509	Pubic symphysis	
6	20510	Humerus	Left, midshaft
6	20511	Iliac crest	
10	20512	Femur	Right, midshaft
10	20513	Rib	Left, midshaft
10	20514	Ulna	Right, midshaft
10	20515	Pubic symphysis	
10	20516	Vertebra	Lumbar, body
10	20517	Humerus	Left, midshaft
10	20518	Ischiopubic ramus	Right
11	20519	Femur	Right, midshaft
11	20520	Rib	Right, midshaft
11	20521	Pubic symphysis	
11	20522	Ulna	Left, midshaft
11	20523	Humerus	Left, midshaft
11	20524	Greater sciatic notch	
12	20525	Femur	Right, midshaft
12	20526 A	Rib	Left, midshaft
12	20526 B	Rib	Left, midshaft
12	20527	Vertebra	Lumbar, body
12	20528	Humerus	Left, midshaft
17	20529	Ulna	Right, midshaft
17	20530	Femur	Left, midshaft
17	20531	Rib	Left, midshaft
17	20532	Vertebra	Lumbar, body
17	20533	Humerus	Left, midshaft
17	20534	Ischiopubic ramus	

**FIGURE 3:**
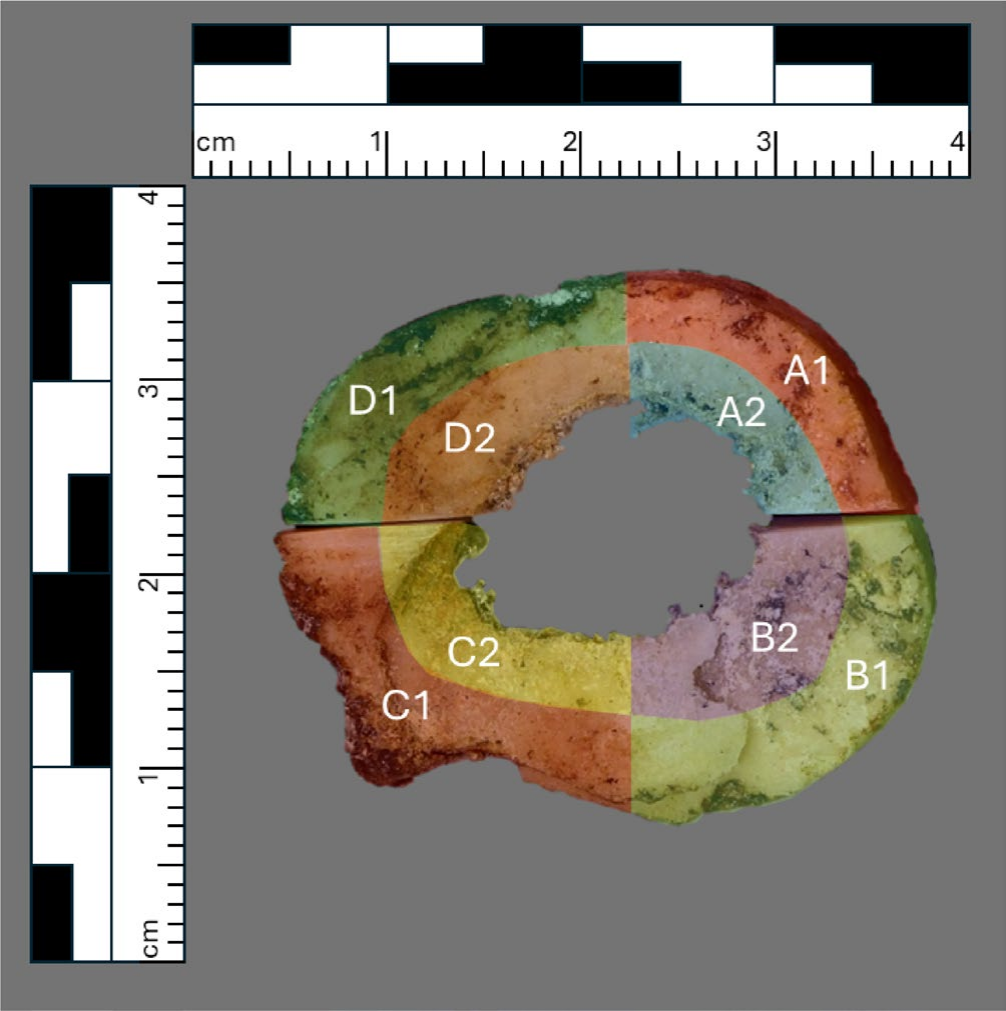
Sampling pattern for one femur (20505) that produced eight subsamples.

For one femur sample (20505), a whole cross section was taken (Figure 3), allowing multiple samples to be taken from a similar region of bone to assess variability in turnover in different areas of the femur. The cross section was subdivided into eight sections, from the medial, lateral, anterior, and posterior area of bone, as well as sampling from the pericortical area and the perimedullary region of bone (Figure 3).

^14^C data from two published studies were collected in order to increase sample size and number of skeletal elements sampled (Johnstone‐Belford et al. 2022; Ubelaker et al. 2022). Details about the samples used in these studies are summarized in the Supporting Information.

### 2.2 Sample Preparation

Two radiocarbon‐dead bone samples and two samples of known Δ^14^C were processed alongside all other samples to determine background ^14^C levels and assess the consistency of sample pretreatments. The collagen isolation process began with lipid removal, which is crucial as lipids contain a significant amount of carbon and likely have a different turnover rate than collagen. Lipid extraction involved 1 h of sonication at room temperature in a 2:1 solution of chloroform:methanol (Folch et al. 1957), repeated twice more with fresh solution for a total of three treatments. This effectively removed lipids (confirmed by elemental analysis), which were then separated and discarded, a necessary step as the ultrafiltration step used during collagen extraction for radiocarbon dating (Brown et al. 1988) may selectively retain lipids rather than remove them (Guiry et al. 2016). Samples were dried at room temperature in a fume hood before demineralization. Demineralization involved placing the samples in 8 mL of 0.5 M hydrochloric acid for 48 h at room temperature while being continuously agitated on an orbital shaker. Complete demineralization was indicated by the samples becoming soft and rubbery, determined by probing with a glass rod. Once demineralization was complete, samples were rinsed to neutrality with Type I water, then 3 mL of 0.01 M hydrochloric acid was added, and the samples were transferred to an oven for reflux at 65°C for 36 h. After refluxing, the solubilized collagen underwent ultrafiltration with Amicon 30 kDa molecular weight cutoff ultrafilters. The solution was centrifuged at 3000 rpm for 20 min, allowing smaller, noncollagenous molecules to pass through the ultrafilter membrane. The remaining solution was collected in 4 mL vials, which were then frozen and lyophilized under vacuum conditions using a freeze dryer.

#### 2.2.1 Isotope Ratio Mass Spectrometry

Prior to AMS radiocarbon dating, stable isotope and elemental analyses were performed on 0.5–0.6 mg aliquots of each collagen sample (except for the ^14^C standards) after weighing the samples into tin capsules. *δ* ^13^C, *δ* ^15^N, %C, and %N values were obtained using a EuroEA 3000 Elemental Analyzer (EuroVector SpA) coupled to a Nu Horizon continuous flow isotope ratio mass spectrometer at the Trent University Water Quality Center.

#### 2.2.2 Analytical Uncertainty

Analytical uncertainty was estimated using the method outlined in Szpak et al. (2017). The random error or *u(Rw*) for the measurements was calculated to be ±0.07‰ for *δ* ^13^C and ±0.21‰ for *δ* ^15^N. This value was derived from replicates of calibration standards, check standards, and sample duplicates. The systematic error, or *u(bias*), was calculated at ±0.09‰ for *δ* ^13^C and ±0.28‰ for *δ* ^15^N based on the difference between the observed and known *δ* values of the standards. The overall analytical uncertainty was calculated to be ±0.13‰ for *δ* ^13^C and ±0.32‰ for *δ* ^15^N.

To assess analytical precision, a 7% sample duplication rate was employed. *δ* ^13^C and *δ* ^15^N values were calibrated relative to VPDB and AIR using a three‐point calibration curve based on USGS40 (*δ* ^13^C = −26.39 ± 0.04‰, *δ* ^15^N = −4.52 ± 0.04‰), USGS63 (*δ* ^13^C = −1.17 ± 0.04‰, *δ* ^15^N = +37.83 ± 0.06‰), and USGS66 (*δ* ^13^C = −0.67 ± 0.04‰, *δ* ^15^N = +40.83 ± 0.06‰) (Qi et al. 2003; Schimmelmann et al. 2016). To ascertain accuracy and precision, in‐house standards of caribou bone collagen (SRM‐1, *δ* ^13^C = −19.39 ± 0.09‰, *δ* ^15^N = +1.85 ± 0.19‰), polar bear bone collagen (SRM‐14, *δ* ^13^C = −13.63 ± 0.09‰, *δ* ^15^N = +21.50 ± 0.22‰), marine fish collagen (SRM‐26, *δ* ^13^C = −16.17 ± 0.10‰, *δ* ^15^N = +14.69 ± 0.18‰), and alanine (SRM‐28, *δ* ^13^C = −16.27 ± 0.09‰, *δ* ^15^N = −1.94 ± 0.18‰) were incorporated into each run. These standards have C:N ratios that closely resemble the anticipated range for human bone collagen (i.e., matrix‐matched), and their *δ* ^13^C and *δ* ^15^N values encompass the upper and lower limits expected for the samples.

#### 2.2.3 Sample Preparation for AMS Radiocarbon Dating

In a quartz tube, 2.0–2.4 mg of collagen from each sample was combusted with 40–50 mg of CuO and silver wire. Radiocarbon‐dead collagen and standard collagen with known age were also combusted in duplicate. These samples were then graphitized at 525°C for 2.5 h. The samples were pressed and loaded into a 0.5 MV NEC‐AMS at the KCCAMS laboratory at the University of California Irvine.

#### 2.2.4 Mathematical Model for Estimating Bone Turnover

The ∆^14^C data generated by AMS can be utilized to estimate the turnover rate of the measured sample. This relies on knowledge of two key factors: the ∆^14^C content of the sample (*C*_measured_) and the atmospheric ∆^14^C content at any year of life (*C*_y_) (Northern and Southern Hemisphere ∆^14^C data are summarized in Table S1) from the year of birth (*b*) to the year of death (*d*). Additionally, we must assign a weight to each *C*_y_, which can also be thought of as the turnover rate (*x*) for each year (_*y*_). It is assumed that turnover rate changes each year, so a specific weight (*x*_y_) can be assigned for each year. For instance, if the element has a decreasing rate of turnover and it decays consistently, with a decay constant (*Z*) of 0.5, the earliest year of life can be weighted as 0.5, the next would be 0.25, the next would be 0.125, and so forth until the year of death. The turnover rate can be applied to the previous year's expected ∆^14^C contents and this continues until the individual's death and their ultimate ∆^14^C value (*C*_expected_). If the *C*_expected_ matches the *C*_measured_, then the model used is a feasible solution for that sample's turnover rates. In this model, there are countless possible turnover scenarios that would lead to the expected ∆^14^C matching the measured ∆^14^C of a sample, as any year's atmospheric ^14^C can be given any weight. However, some of these solutions are clearly illogical and, therefore, only models that are consistent with the current understanding of bone formation and loss over life should be considered. Therefore, a decaying turnover rate model was applied. It provides a static decay constant (*Z*) which determines each annual turnover rate and closely resembles current understandings of how turnover may change over life, specifically that it is highest early in life and much lower with advanced age (Feik et al. 1996; Frost 1997; Fahy et al. 2017; Hedges et al. 2007). This decaying turnover rate is mathematically expressed using Equation (1) and assumes that *x*_0_ = 100% since all bone must have been formed within the year of being in womb.

(1) $$x_{y}=\left(x_{y}-1\right)\left(1-Z\right)$$

Determining the “correct” decay constant for a sample involves an iterative process of testing various decay constants within the fluctuating turnover equation (Equation 2) until the *C*_expected_ matches the *C*_measured_ of that sample.

(2) $$C_{\text{expected}\;\left(y\right)}=\left(1-Z^{a}\right)\times C_{\text{expected}\;\left(y-1\right)}+C_{y}Z^{a}$$

Here, *C*_expected (*y*)_ refers to the estimated Δ^14^C content that the individual would have had at any year of life (*y*). Z refers to the decay constant, *a* refers to the age of the individual in years at the desired year, and *C*_y_ refers to the atmospheric Δ^14^C content within that year. This equation can be worked backwards until birth (*b*), where *C*_expected(*b*)_ is thought to be equal to *C*_y._

### 2.3 Hedges et al. (2007) Model

**FIGURE 4:**
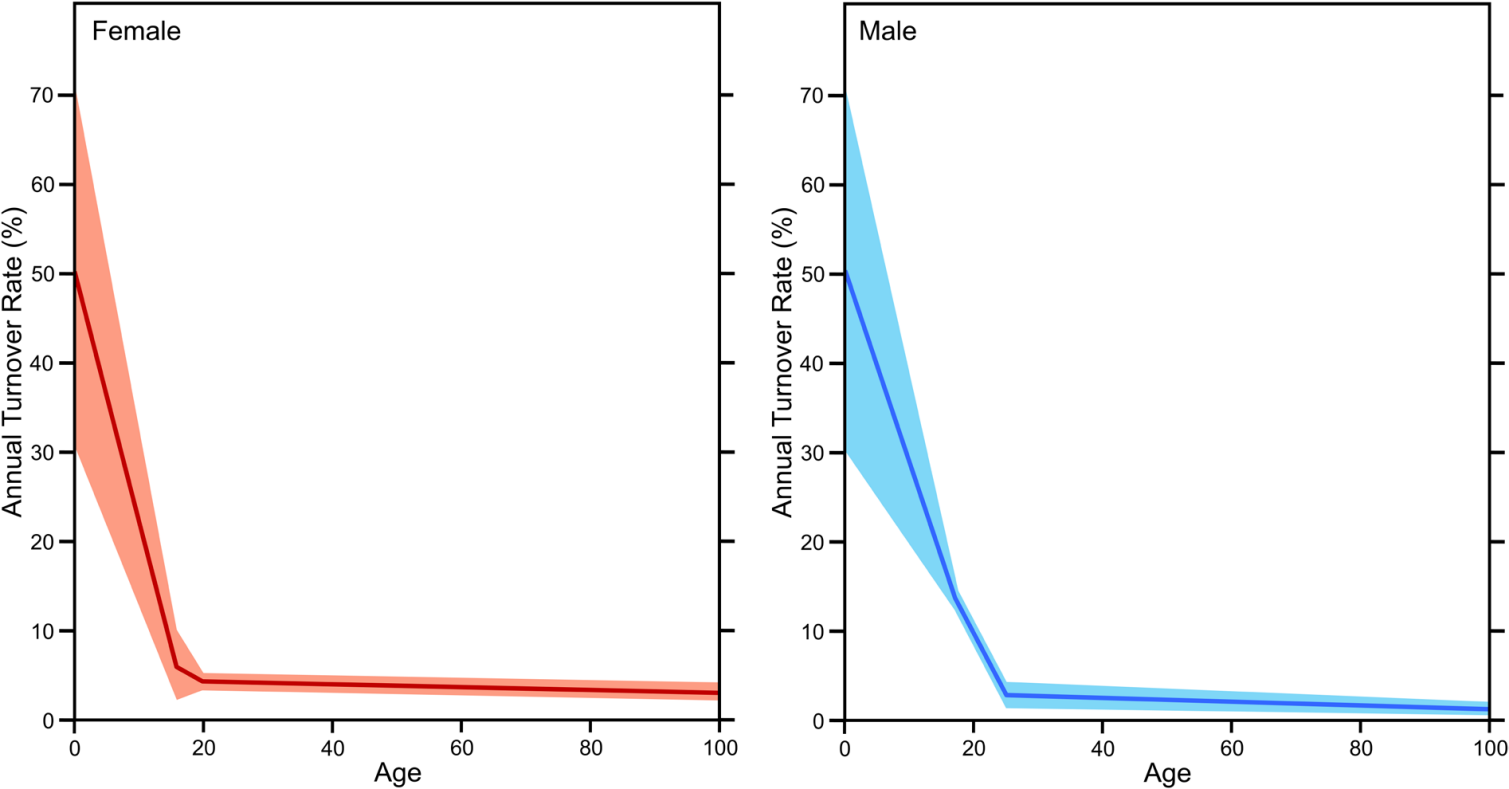
Annual turnover rates at each year for the femur according to the model of Hedges et al. (2007). Females are on the left and males are on the right. The solid lines represent the means, and the shaded areas represent the ± value given by Hedges et al. (2007).

An alternative model presented by Hedges et al. (2007) has produced the estimated turnover interval most frequently cited in stable isotope literature. Hedges et al. established a sex‐specific turnover model, employing a turnover pattern with age‐linked inflection points (Figure 4). The general shape of the curve broadly resembles the exponential decay function discussed previously but is more linear in nature. This approach involved delineating inflection points where the turnover rate transitioned, gradually decreasing each year until reaching the subsequent inflection point. The turnover rates were derived from an average best fit for the sampled femora. The specific values used to generate the curves are presented in Table 3.

**TABLE 3:** The model used by Hedges et al. (2007) with varying turnover rates based on sex and age.

Inflection point	Age (males)	Annual turnover rate (%)	Age (females)	Annual turnover rate (%)
Birth	0	50 ± 20	0	50 ± 20
Adolescence	17	13.7 ± 1.3	15	6 ± 4
Cessation of growth	25	3 ± 1.5	19	4 ± 1
Death definitely occurred	100	1.5 ± 0.7	100	3 ± 1

We tested the Hedges et al. (2007) turnover model by comparing the REST[ES] femur sample's modeled Δ^14^C against the real measured Δ^14^C of each femur sample. We used the average as well as both upper and lower limits of turnover rates at each inflection point (Figure 4) to generate several different modeled Δ^14^C for each sample. The expected and measured Δ^14^C were statistically compared to determine if the Hedges et al. (2007) accurately predicted the Δ^14^C values of the REST[ES] femora.

### 2.4 Data Treatment

The results of the stable isotopic analysis were used to determine if a significant portion of each individual's diet came from marine resources, to assess whether the marine reservoir effect influenced the samples' Δ^14^C values. The ocean experienced a more dampened increase in ^14^C content relative to the atmosphere during the period of above‐ground nuclear weapons testing (Dutta 2016). Thus, a diet high in marine protein could produce inaccurate turnover estimates. Modern Canadian diets are largely homogeneous due to food industrialization, with appreciable marine protein consumption being rare, except in some Inuit communities (Bataille et al. 2020). On this basis alone, it is unlikely that the donors at the REST[ES] facility who all lived in Quebec were influenced by a marine effect. However, identifying a range indicative of a highly marine‐influenced diet can still be useful. Schoeninger and DeNiro (1984) suggested that bone collagen *δ* ^13^C values of −16.5‰ or higher, along with *δ* ^15^N values of +13.6‰ or higher, indicate a high marine diet. An isotopic study of fingernails from the Uummannaq Inuit population of Greenland, who primarily consume marine protein, found values above +12‰ for *δ* ^15^N and above −20‰ for *δ* ^13^C (Buchardt et al. 2007); this latter value would likely be approximately −18.6‰.

The Δ^14^C values were substituted into Equation (2) to estimate the turnover rate of the sample through an iterative process, attempting to match the *C*_measured_ with the *C*_expected_ by varying the number of years in the turnover interval and the relative weighting of each year. All Δ^14^C distributions were tested for normality using a Shapiro–Wilk test. For comparisons between two groups (e.g., males and females), an *F*‐test assessed the equality of variances, followed by a *t*‐test (if variances were equal) or Welch's *t*‐test (if variances were unequal). If distributions were non‐normal, a Mann–Whitney *U* test was used. For comparisons involving three or more groups, a one‐way ANOVA was performed if all distributions were normal, followed by Levene's test for equality of variances. If variances were equal, a post hoc Tukey's HSD test was used; if unequal, a Dunn's post hoc test was applied. A Kruskal–Wallis test replaced the ANOVA when one or more groups had a nonparametric distribution.

To estimate the percentage of each sample attributed to different periods of an individual's life, a Python script was employed using Google Colaboratory to model bone tissue changes based on the resulting turnover rate for the decaying model. The Python script was generated with AI assistance (Google 2024) and is shown in script 1 of the Supporting Information. This model uses a selection of 100 units of bone and randomly replaces a specified percentage of the bone units based on annual turnover rates for each year, continuing this process until each individual's age at death. At death, each unit of bone in the model indicates the last year it was “turned over,” and these years were used to calculate the percentage of units from each life period in the sample. Due to the random changes in bone units, variation exists between runs of the same model; thus, for each sample, the model was run 1000 times, and the average and standard deviation of the 1000 runs for each decade of life were recorded.

## 3 Results and Discussion

### 3.1 Stable Isotopes

The stable isotope data for all bone collagen samples analyzed in this study are summarized in Table S2. Quality control criteria for 46 out of 53 samples met the most stringent acceptable atomic C:N ratio range of 3.00–3.28 for modern bone collagen (Guiry and Szpak 2020). Six samples had C:N ratios between 3.29 and 3.32, and one sample had a ratio of 3.46. There was a statistically significant negative correlation between the C:N ratios and *δ* ^13^C values for these samples (Pearson's *r* = −0.45, *p* < 0.001). Excluding samples with C:N ratios above 3.28 still resulted in a significant correlation between C:N and *δ* ^13^C (Pearson's *r* = −0.32, *p* = 0.03). This suggests that some samples with higher C:N ratios may contain residual lipids not removed by ultrafiltration (Guiry et al. 2016) or the chemical lipid extraction, but the impact was minimal, as indicated by the low variation in *δ* ^13^C values. Elemental concentrations (%C and %N) were consistent with expectations for modern mammalian bone collagen (Guiry and Szpak 2020). All *δ* ^13^C values were above −20‰, likely due to a diet with a relatively high amount of protein from maize‐fed animals (Jahren and Kraft 2008). No *δ* ^15^N values exceeded +12‰, indicating a negligible contribution from marine foods. Therefore, any Δ^14^C values obtained were not significantly affected by the marine reservoir effect.

### 3.2 Decay Constants and Skeletal Element Differences

**FIGURE 5:**
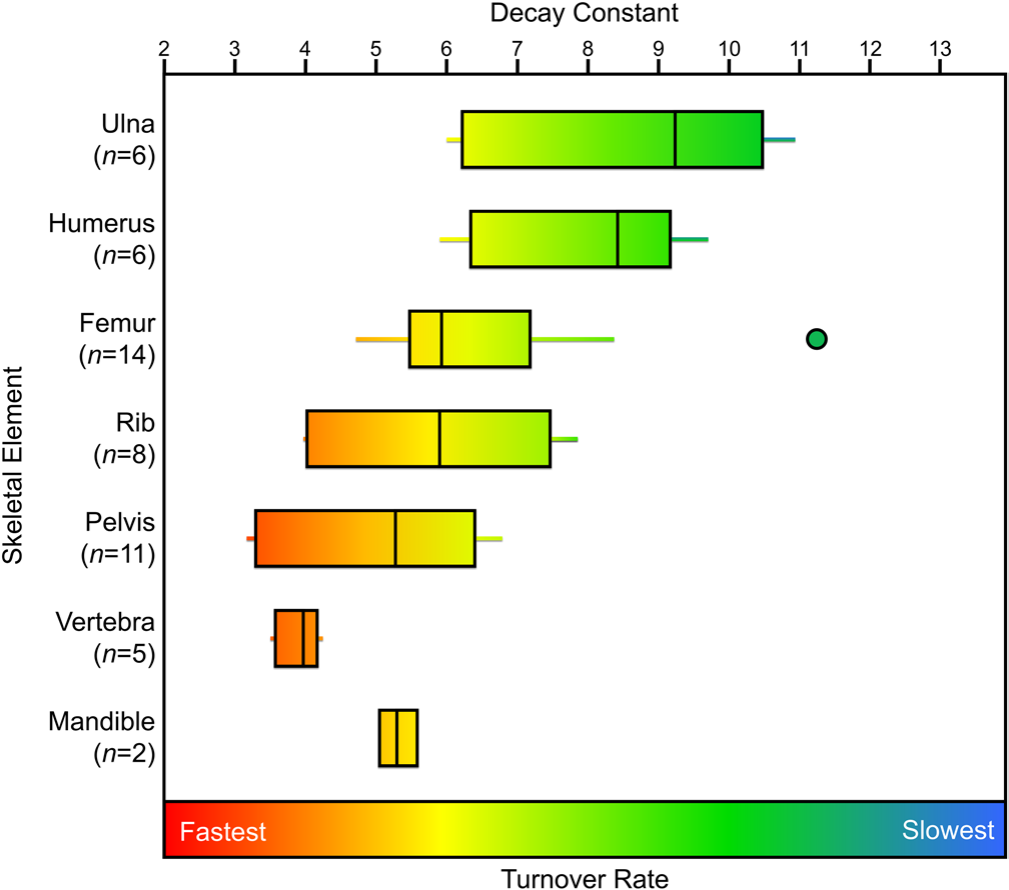
Box plots representing the decay constants (a proxy for bone turnover rate) calculated for skeletal elements from all REST[ES] donors.

The decay constants and the estimated percentage of tissue synthesized in each decade of life for each of the skeletal elements of the REST[ES] donors are presented in Table S3. The ulna typically had the highest decay constant (slowest turnover rate) but with considerable variation. The vertebra had the lowest decay constants (highest turnover rates) with relatively low variation among the five samples analyzed (Figure 5). Apart from the vertebra, each element had a large range of estimated decay constants and hence turnover rates. A Kruskal–Wallis H test demonstrated that there were significant differences in the decay constants and hence turnover rates among skeletal elements (*χ* ^2^ = 23.03, *p* ≤ 0.001), with the vertebra decay constant being significantly different than the ulna (*p* < 0.001), humerus (*p* < 0.001), femur (*p =* 0.005), and rib (*p =* 0.03). The pelvis decay constant was also different than the ulna (*p =* 0.002) and humerus (*p* = 0.009), and the rib was different than the ulna (*p* = 0.035). Notably, the decay constants of the femur and rib were not significantly different.

**FIGURE 6:**
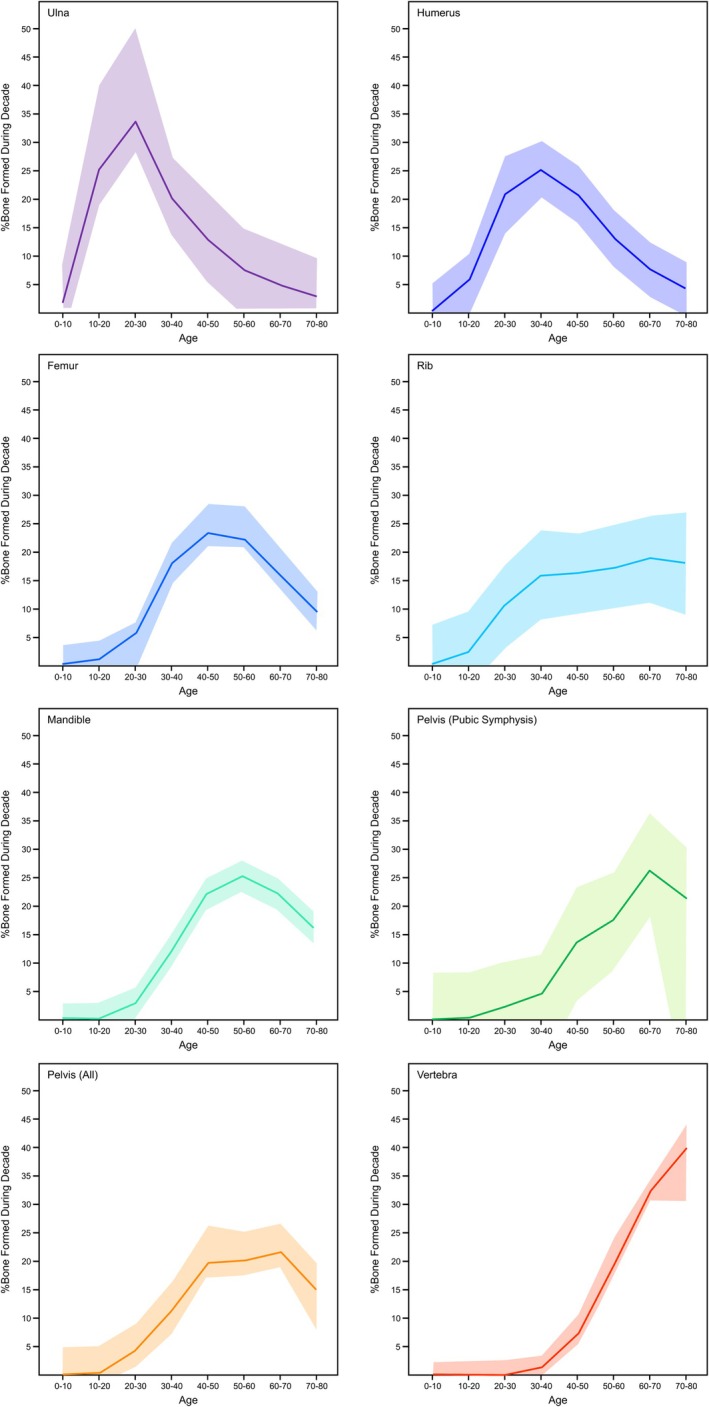
Estimated percentage of bone formed in each decade of life for the seven elements analyzed from the REST[ES] donors if the samples were taken at age 80. The data are represented through a locally estimated scatterplot smoothing (LOESS) curve. Note that the pelvis is represented twice, once for the pubic symphysis (the most frequently sampled site; five donors) and once including all sampled sites on the pelvis (pubic symphysis, iliac crest, greater sciatic notch).

Figure 6 shows the average percentage of bone collagen that was produced in each decade of life across the elements analyzed from all donors, with the average age of death for these individuals being 71 years old. To account for the variable age at death for these donors, bone turnover was projected to the age of 80 for all donors.

**FIGURE 7:**
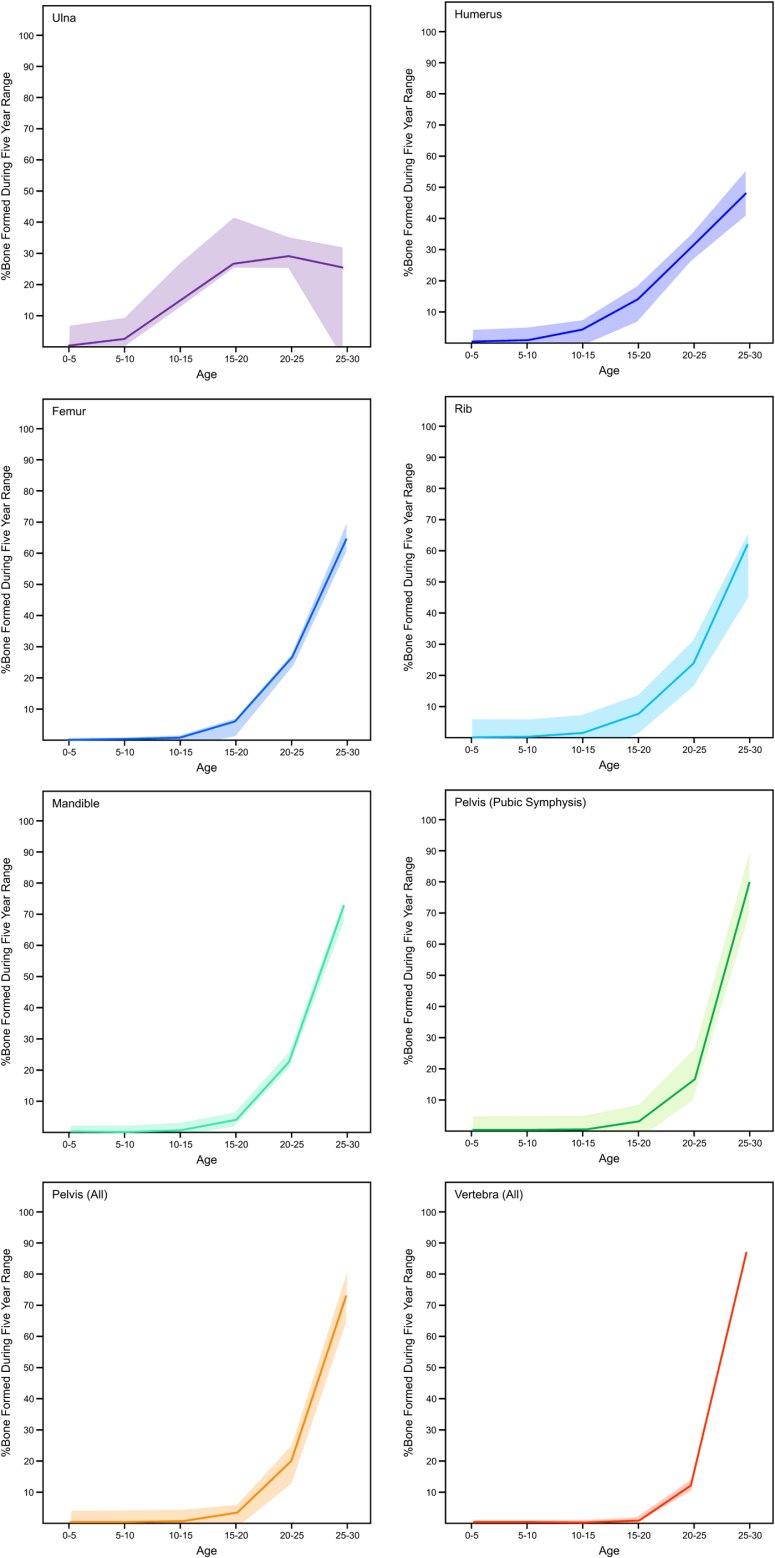
Estimated percentage of bone formed in each decade of life for the seven elements analyzed from the REST[ES] donors if the samples were taken at age 30. The data are represented through a locally estimated scatterplot smoothing (LOESS) curve. Note that the pelvis is represented twice, once with the pubic symphysis (the most frequently sampled site; five donors) and once including all sampled sites on the pelvis (pubic symphysis, iliac crest, greater sciatic notch).

For the older individuals included in this study, the ulna and humerus are better suited to reflect diet and activities from ages 0 to 40, whereas the pelvis and vertebra are more heavily weighted toward age 40–80. The femur was more evenly weighted throughout each decade; however, it is most accurately reflected between age 30 and 70. It is important to note that many of these estimates have a relatively high degree of variance due to sometimes very different ^14^C values among individuals. These interelement differences are far less pronounced at age 30, where all skeletal elements should primarily reflect the last 5 years of life. There are, however, still substantial interskeletal differences in turnover rates. The ulna and humerus contain much more collagen formed in adolescence than bones such as the pelvis and vertebra. Since we did not measure any individuals that died around age 30, the estimates presented in Figure 7 are associated with much more uncertainty than those presented in Figure 6.

**FIGURE 8:**
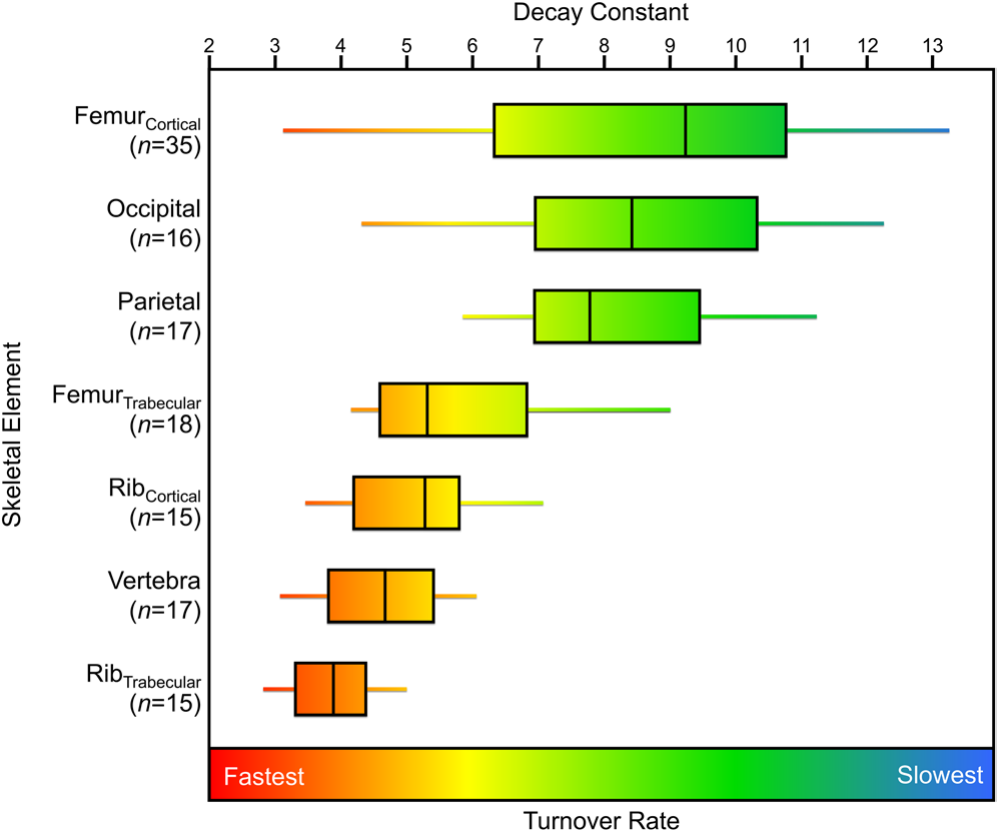
Box plots representing the decay constants (a proxy for bone turnover rate) calculated for skeletal elements from two previously published southern hemisphere studies (Ubelaker et al. 2022; Johnstone‐Belford et al. 2022).

The ^14^C results and decay constants from the individuals from Ubelaker et al. (2022) and Johnstone‐Belford et al. (2022) are presented in Tables S4 and S5, with the percentage of bone collagen attributed to each decade of life for these individuals presented in Table S6. Differences between each skeletal element's turnover rates (as represented by decay constants) are summarized in Figure 6. A Kruskal–Wallis test of the individuals from Ubelaker et al. (2022) and Johnstone‐Belford et al. (2022) indicated that there were differences among the estimated decay constants of the skeletal elements (*χ* ^*2*^ = 54.56, *p* < 0.001); the results of Dunn's post hoc tests are presented in Table 4. Of the skeletal elements used in these datasets, the trabecular rib generally had the highest turnover rate (lowest average decay constant, 4.0%), which was significantly less than all other elements except the vertebra and cortical rib (Table 4). The cortical femur had the largest range in turnover rates (decay constants ranging from 13.3% to 3.2%), also having the lowest turnover rate (highest average decay constant of 8.8%) (Figure 8). The parietal and the occipital also had relatively low turnover rates (high decay constants), slightly higher than but not significantly different from the cortical femur (Figure 8, Table 4). These other studies separated cortical and trabecular sections of the femur and rib, but only the femur had a significantly different turnover rate between these bone types (Table 4).

**TABLE 4:** Results of Dunn's post hoc tests (*p* values) for differences in decay constants for each skeletal element group.

	Occipital	Femur_Cortical_	Femur_Trabecular_	Rib_Cortical_	Rib_Trabecular_	Vertebra
Parietal	0.95	0.88	**0.015**	**< 0.001**	**< 0.001**	**< 0.001**
Occipital		0.82	**0.019**	**0.001**	**< 0.001**	**< 0.001**
Femur_Cortical_			0.006	**< 0.001**	**< 0.001**	**< 0.001**
Femur_Trabecular_				0.43	**0.048**	0.26
Rib_Cortical_					0.22	0.82
Rib_Trabecular_						0.26

*Note:* Statistically significant differences (*p* < 0.05) are indicated with boldface.

**FIGURE 9:**
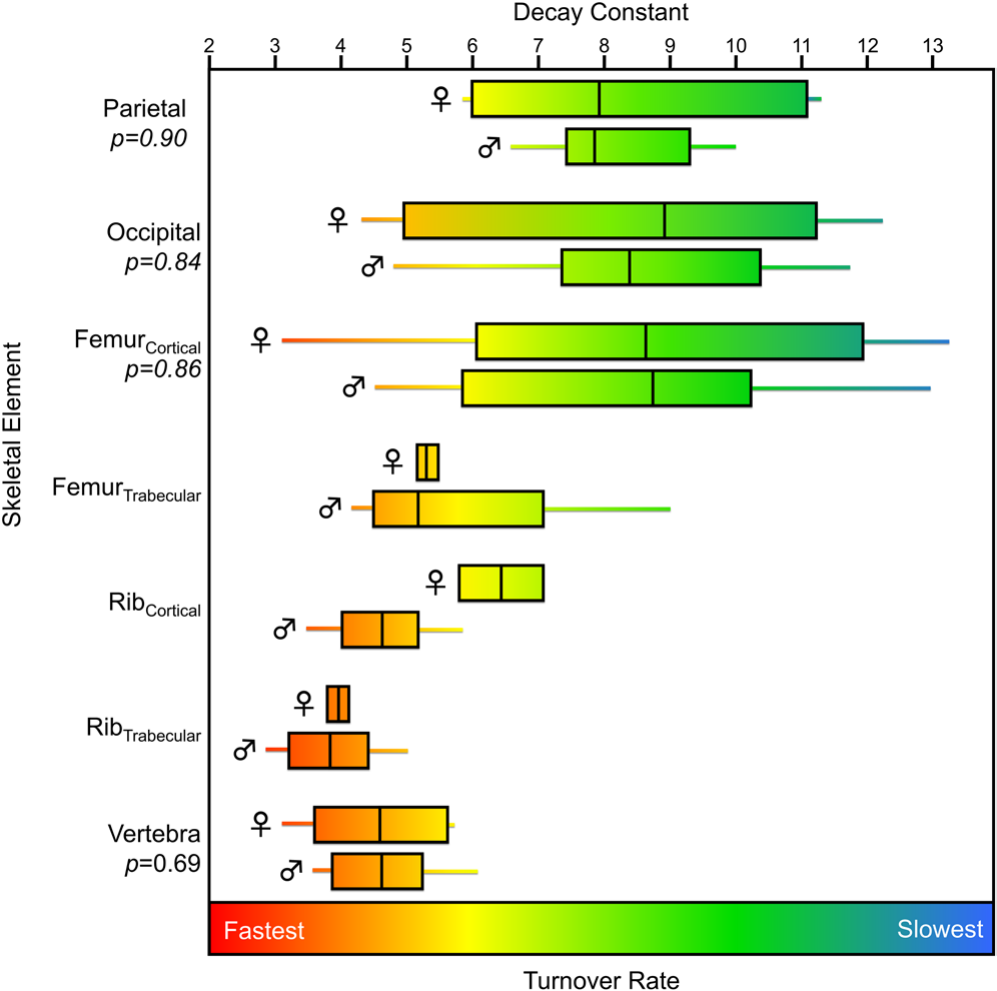
Decay constants (a proxy for bone turnover rate) for the different skeletal elements analyzed in the two southern hemisphere datasets (Ubelaker et al. 2022; Johnstone‐Belford et al. 2022) by sex (identified by ♀ and ♂). *P* values for statistical tests are indicated (all unpaired *t*‐tests except for the vertebra, which was a Mann–Whitney *U* test) for those elements for which a sufficient number of specimens were present.

The low sample size of females in the REST[ES] dataset made it so statistical analysis comparing the male and female donor decay constants were not possible. General comparisons of the same skeletal elements from REST[ES] male and female donors suggested no differences between the sexes in bone collagen remodeling rates, but the small sample size precludes any conclusions from being drawn. The Southern Hemisphere datasets (Ubelaker et al. 2022; Johnstone‐Belford et al. 2022) had a larger number of individuals of each sex, which allowed for statistical analysis to be performed. The results show that no significant differences in decay constant were found between the sexes for any skeletal element group (Figure 9), however, the trabecular femur as well as the two rib categories did not have enough female data for statistical analysis. While it is a small sample, these results agree with lack of sex‐based differences in bone collagen turnover observed in the REST[ES] dataset.

### 3.3 Hedges et al. (2007) Model

**FIGURE 10:**
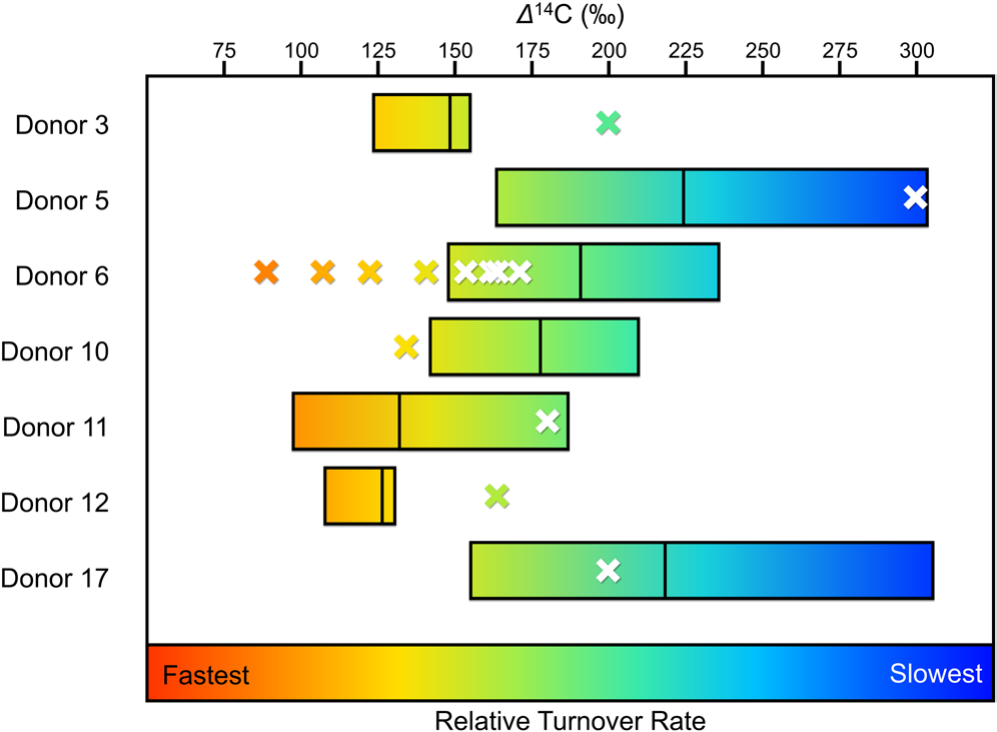
Estimated Δ^14^C values for REST[ES] donors based on upper and lower limits of the femoral bone collagen turnover model presented by Hedges et al. (2007), represented as boxes. The measured Δ^14^C values for the REST[ES] donors are indicated with an ×. The color scale in this figure does not correspond to the turnover rates or decay constants in previous figures.

The Hedges et al. (2007) study predicted inflection points and upper and lower turnover rates for male and female femora (Table S7). If accurate, the measured Δ^14^C values from the REST[ES] femora should align with the predicted Δ^14^C ranges based on the Hedges et al. (2007) model. Figure 10 shows the overlap between the estimated and measured Δ^14^C values. The estimated Δ^14^C range (using the lower and upper ends of the Hedges et al. (2007) model) failed to predict the actual Δ^14^C in approximately half the samples. This indicates that the estimated turnover rates at every inflection point for the femur predicted by Hedges et al. (2007) may be inaccurate for any person and are not reliable.

### 3.4 Relative Turnover Rates Among Skeletal Elements

This study reveals a general ordering of skeletal elements by turnover rate from slowest to fastest (Table 5). In the REST[ES] dataset, the ulna consistently showed the slowest turnover, followed by the humerus, femur, rib, pelvis, and the vertebra, which had the fastest turnover. Although the mandible's small sample size limits the confidence that we should have in its turnover rate relative to other more well‐represented elements, it aligned closely with the rib and femur, suggesting a similar turnover rate; however, more data are needed to confidently identify the mandible's relative turnover rate. Applying a similar data modeling approach to ^14^C data collected from two other studies (Ubelaker et al. 2022; Johnstone‐Belford et al. 2022) indicated that while the turnover rate of the rib was significantly higher than the femur, the turnover rates of the rib and femur were broadly similar to one another (Figures 5, 8, and 9). This pattern is consistent with the very similar osteon population density counts observed by Fahy et al. (2017), suggesting similar turnover rates. The occipital and parietal showed slow remodeling rates, similar to the femur in the Southern Hemisphere dataset, but these two cranial bones had much slower turnover rates than the femora from the REST[ES] donors. For all three datasets that were examined, the vertebra had the fastest turnover rate among the skeletal elements that were analyzed.

**TABLE 5:** Qualitative order of skeletal elements from slowest remodeling to fastest bone collagen remodeling.

Slowest turnover rate	Ulna
	Humerus
	Femur
	Rib
	Mandible^a^
	Pelvis
Fastest turnover rate	Vertebra

^a^ This bone had a small sample size and therefore this ranking is uncertain.

### 3.5 Factors Influencing Bone Turnover

Previous studies have suggested various factors that are associated with variable bone turnover rate, which might explain why there is a high degree of variation in estimated turnover rate within a skeletal element (Figures 5, 8, and 9). Such factors have included strain or mechanical loading (Huiskes et al. 2000; O'Connor et al. 1982; Sykut et al. 2020; Skedros et al. 2013), spatial differences in bone turnover independent of mechanical loading (de Gruchy et al. 2024; Hall et al. 2024; Ubelaker et al. 2006), sex and age (Feik et al. 1996; Mulhern 2000; Fahy et al. 2017; Han et al. 1997), ethnicity (Han et al. 1997; Weinstein and Bell 1988), and health status (Parfitt 1996; Ingle et al. 1999; Shigdel et al. 2015; Melton et al. 1997).

**FIGURE 11:**
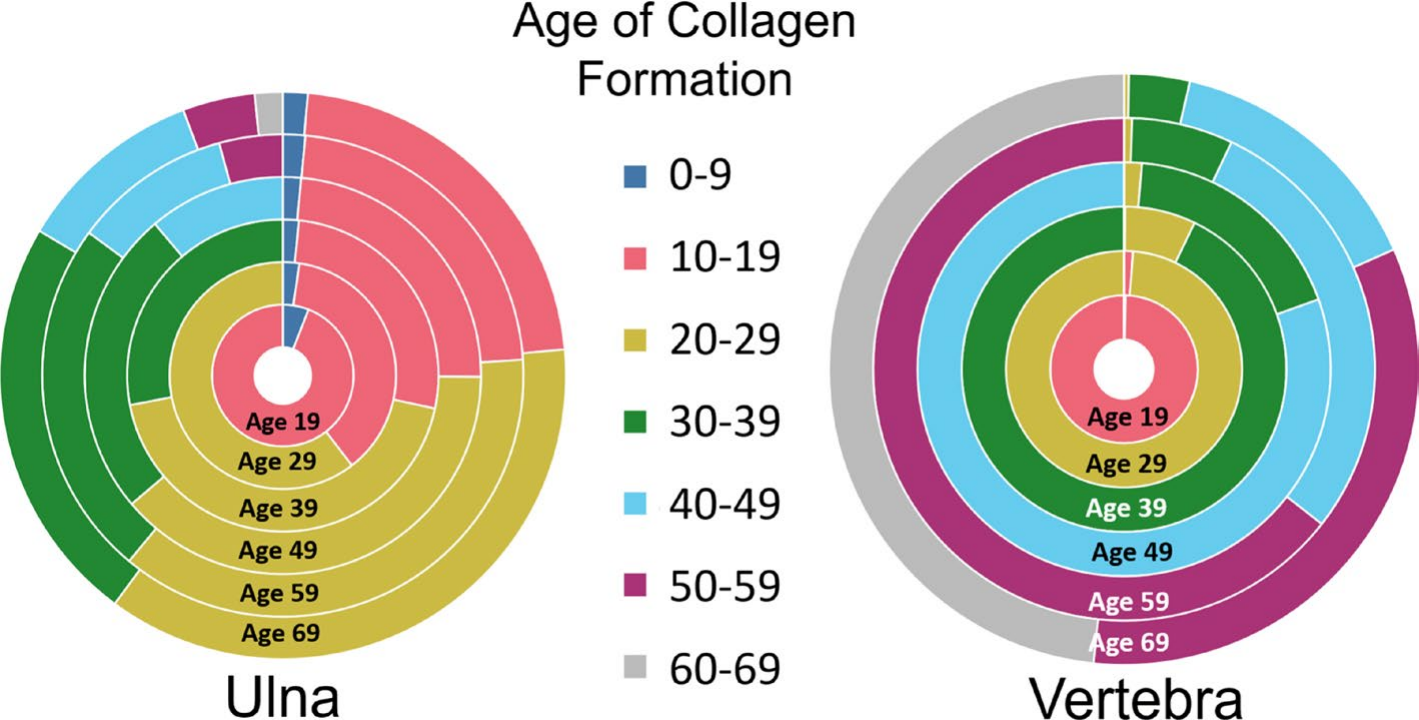
The percentage of a bone collagen sample that would be attributed to each decade of life from the Ulna and the Vertebra of REST[ES] Donor 3 across different ages of life. The innermost ring represents the estimated sample composition of the individual at age 19, and the outermost ring represents the composition at death (age 69). Data are produced using a “random remodeling” scenario (Data are produced assuming no sections are preferentially remodelled and remodelling is truely random).

Age at death plays a critical role in determining the relative age composition of collagen as the turnover rate changes with age (Bryant and Loutit 1963; Fahy et al. 2017; Hedges et al. 2007). Figure 11 illustrates how the percentage of bone collagen attributed to each decade of life shifts across different ages. In a bone with a slow rate of collagen turnover (e.g., ulna), the composition remains relatively stable, with the percentage of bone from young adulthood staying similar as the individual ages. In contrast, bones with faster turnover rates like the vertebra replace collagen produced in earlier decades more quickly so that at any given age, most of the bone has been formed within the preceding decade. Age is often overlooked when discussing bone collagen turnover rates in stable isotope studies. While adults and subadults are sometimes distinguished, most research treats all adults as a homogenous group (e.g., Fernández‐Crespo et al. 2022; Gaydarska et al. 2025; Pickard and Bonsall 2022). Knowing an individual's age is crucial when determining specific turnover rates, but such estimates are challenging, especially when remains are fragmentary or poorly preserved. In these cases, it is essential to acknowledge and communicate the uncertainty of the turnover rates.

Collagen turnover rates are likely influenced by the mechanical loading placed on the bone. Frequent, low‐impact loading has been observed to stimulate remodeling (Bergmann et al. 2010; Turner et al. 1995), therefore, bones subjected to more regular loading may be associated with faster turnover rates (lower decay constants). Bones subject to regular loading, like the pelvis and vertebra, tend to have faster turnover rates, while those experiencing less force, such as the ulna and humerus in typical modern humans, remodel more slowly (Figure 6). Ribs, which undergo low strain loading during respiration, have moderate collagen turnover rates. Cranial bones, facing minimal stress, exhibit some of the slowest rates observed (Figure 8). Activities influencing lower limb loading tend to vary between people since activity rates are highly variable, likely contributing to the high variability in femur turnover. Differences in estimated bone collagen turnover rates for the femur between the REST[ES] and Southern Hemisphere datasets may reflect varying activity levels, though the precise reasons remain unclear due to a lack of data concerning the degree of mobility or physical activity of these individuals. Donor 17, paralyzed in his final 7 years of life, offers insight into how reduced movement may affect turnover. His overall decay constants were higher than average, indicating slower turnover rates, particularly in the pelvis and ulna. This could be due to increased osteoclastic activity in trabecular bone and the selective removal of newer bone near the medullary cavity (Ausk et al. 2017; Bergmann et al. 2010; de Gruchy et al. 2024). These findings suggest a link between bone loading, activity levels, and turnover rates across skeletal elements.

Any number of health factors could influence the results of this study, and with a small sample size, it is hard to separate health factors from what “normal” turnover may be. For example, an interesting result was that the donor who took alendronate for osteoporosis had the lowest decay constants, indicating the highest turnover rates in her humerus, rib, and vertebra compared to the same elements from other donors in this study. Alendronate has been shown to increase collagen production (Boanini et al. 2008; Tsuchimoto et al. 1994), which may explain the higher turnover rates observed in this donor. If other donors experience the effects of osteoporosis, it is thought that this disease alone should not affect the ^14^C results observed here. An increase in osteoclastic activity associated with the disease (Garnero and Delmas 1998), while decreasing bone mass, should remove collagen randomly from the bone thus maintaining the Δ^14^C values. It is also important to note that a large percentage of the donors in this study had some form of cancer. While no donor specifically had cancer of the bone, melanoma and lung cancer can both cause osteolytic metastasis (Roato et al. 2010; Hiraga et al. 1995), which would result in lesions on the bone. Since no lesions were observed during sampling, and since osteolytic metastasis should remove bone tissue randomly (as it relates to the ^14^C content of the collagen), it is unlikely that the effects of these instances of cancer on bone affected the results of this study. Importantly, multiple other health factors such as illness in childhood, malnutrition or other nutritional deficiencies, and other non‐visible health factors may influence bone turnover, potentially driving the high level of variation observed within the skeletal elements sampled in this study.

Although the REST[ES] dataset had a limited number of female donors, the Southern Hemisphere data provided better female representation for comparison of sex‐based differences. Surprisingly, the decay model revealed no discernible differences between male and female samples, despite commonly held assumptions of sex‐based differences in bone remodeling (e.g., Hedges et al. 2007). Quinn (2023) noted negligible differences found between the turnover rates of femora for males and females based on their modeling of several published datasets (Hedges et al. 2007; Ubelaker and Parra 2011; Ubelaker et al. 2022; Johnstone‐Belford et al. 2022). Physiologically, one might expect differences due to girls typically starting puberty earlier than boys (Rosenfield et al. 2000), and hormonal changes later in life that could influence remodeling (Feik et al. 1996). However, this study found no evidence to support these differences, suggesting that individual variability in turnover rates among skeletal elements may overprint any sex‐based differences. It is also possible that these remodeling differences are obscured with advanced age, precluding their detection with the REST[ES] dataset. A more comprehensive analysis with a larger sample and broader age range (especially younger and middle‐aged individuals) is necessary to confirm this finding of parity in bone collagen turnover rates between males and females.

This study sampled different locations on elements such as the rib and mandible, various portions on the pelvis, whole cross sections of the femur, and different vertebrae (lumbar and thoracic), revealing varying degrees of variation within skeletal elements. Turnover rates between neighboring bone regions were generally more similar than those within the femoral cross section. Samples from this cross section (Figure 3) had the second highest and third‐lowest decay constants of this donor's skeletal elements. No clear pattern emerged regarding sampling location, possibly due to the coarse resolution. Differences between cortical and trabecular bone were also visible, with trabecular bone generally having higher turnover rates, aligning with prior findings (Bryant and Loutit 1963; Hill 1998; Cox and Sealy 1997). Within the pelvis, variable turnover rates based on sampling location were noted, with the iliac crest having higher average decay constants (lower turnover rates) and the pubic symphysis being lower (higher turnover rates), likely due to differences in trabeculation and physical demands (Zaharie and Phillips 2018). Lumbar and thoracic vertebrae comparisons showed thoracic vertebrae had slightly higher decay constants (slower turnover rates), possibly due to lower loads on the spine. Overall, while predicting relative turnover rates based on bone type (cortical or trabecular) is possible, sampling location can also influence results in a large, seemingly unpredictable manner. The intrabone variation in estimated turnover rate supports this notion. The variable location of sampling due to constraints surrounding the conditions of the donors could have resulted in the slightly different sampling locations, reflecting different temporal compositions of bone based on sample location alone rather than differences between donors. This underscores the importance of identifying sample locations in isotope studies, when possible, though predicting exact turnover rates remains challenging without ^14^C measurements at high spatial resolution within skeletal elements.

### 3.6 Conceptualizing Turnover

It is important to emphasize that we do not assume that turnover rates uniformly follow a decaying turnover model throughout life. To compare different skeletal elements and individuals effectively, some generalizations are necessary for simplification. This was particularly crucial in this study, as all participants were from one age category, limiting the ability to determine specific turnover rates at each age. The decay model was selected because it broadly resembles past models (Hedges et al. 2007; Quinn 2023), showing high turnover in early life followed by a rapid decline post‐growth. Numerous studies indicate that bone formation decreases with age due to reduced muscle use and overall loading on bones (e.g., Feik et al. 1996; Han et al. 1997; Fahy et al. 2017; Frost 1997; Birkhold et al. 2014). In this model, the turnover rate at any age can be estimated after determining the decay constant on the basis of the ^14^C content at death. Although bone turnover rates are higher in early life than in old age, accurately determining these rates using ^14^C measurements from older individuals is difficult because many bones lack tissue that was formed in these early years since it has been replaced through turnover. In this study, some samples had no bone left that was formed during primary growth, making estimates of turnover rates from these years unreliable as these rates entirely rely on the decaying model to estimate the turnover. However, for bones like the ulna, which must contain some early life bone collagen (the very high Δ^14^C values are otherwise impossible to explain), estimates of turnover rates earlier in life should be more accurate, though still associated with a high degree of uncertainty. While average annual turnover rates can be confidently estimated for older adults in this study, accuracy decreases for early life rates across all skeletal elements, especially those with higher turnover rates (i.e., vertebrae and pelvis).

### 3.7 Implications for Future Studies

**FIGURE 12:**
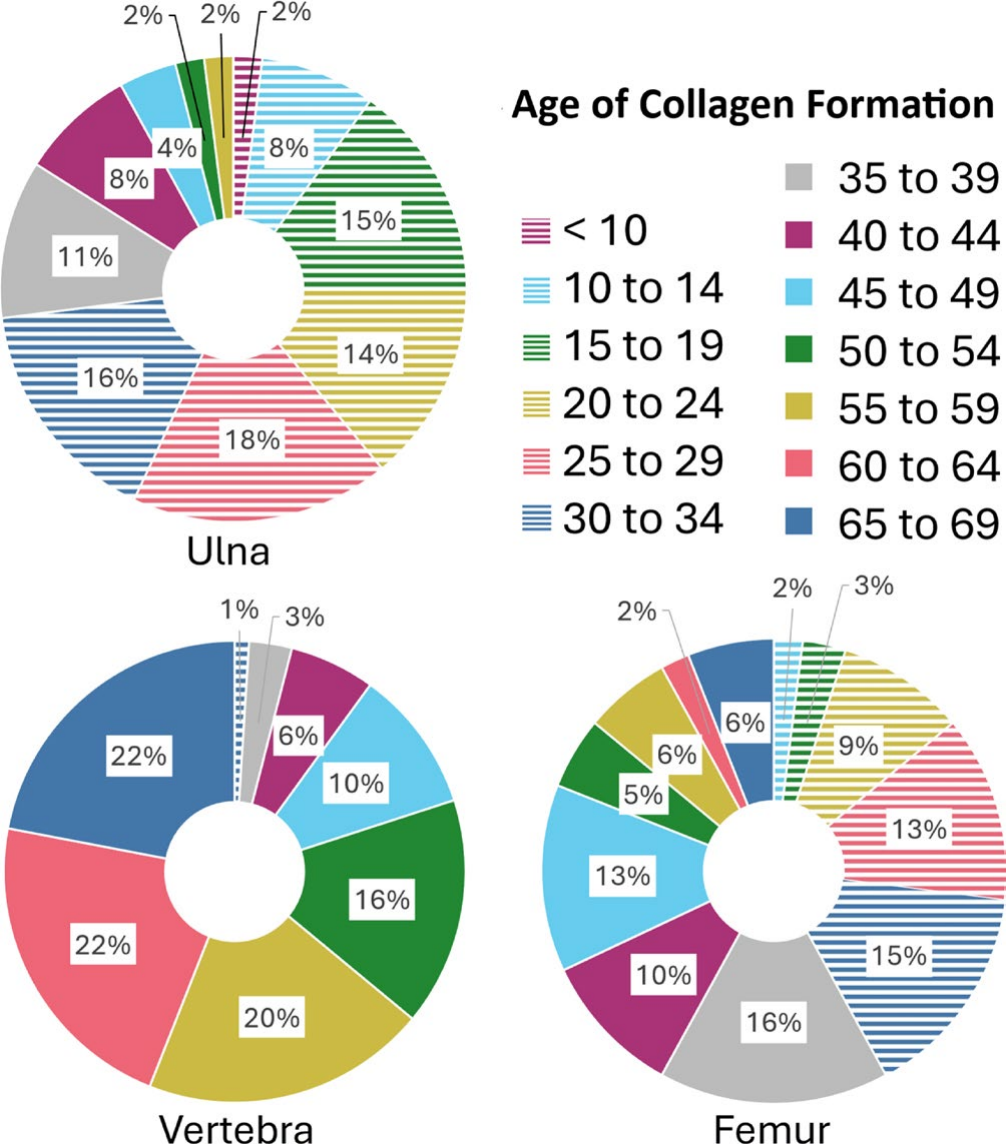
The percentage of a bone sample at death attributed to each age range from the femur, vertebra, and ulna from Donor 3, who died at age 69. The first 35 years of life are represented by areas filled with horizontal lines, while the last 35 years are represented by solid fills.

Stable isotope researchers frequently use bone collagen in order to assess the diet and life history of archeological individuals. The period of life represented by the isotopic compositions of these bones is incredibly important to draw proper conclusions, particularly in those studies that make diachronic inferences about the lives of individuals (e.g., Cox and Sealy 1997; Cheung et al. 2017; Lamb et al. 2014). Based on the results of this study, there are clearly vast differences in the period of life that each bone collagen sample's isotopic composition will represent. It is, therefore, critical for researchers to focus their sampling efforts on elements for which collagen turnover patterns have been adequately characterized. In many cases, especially for cranial and long bones, collagen samples are likely to contain more information from an individual's adolescence than one might expect, with less collagen reflecting the years immediately before death as the individual ages. Although this notion has previously been communicated (Matsubayashi and Tayasu 2019), it has rarely been adequately considered in stable isotope research. This concept is visualized in Figure 12 on the basis of the ^14^C data from REST[ES] Donor 3, with the ulna having a collagen turnover interval reflecting almost the whole life but disproportionately weighted to the first 35 years of life. The vertebra is characterized by the opposite pattern, with the collagen being formed primarily in the last years of life, with collagen forming in the first 35 years of life being almost completely replaced. The femoral collagen from this donor is somewhat intermediate between these two. Turnover intervals are typically used to characterize the period of time represented by the isotopic composition of bone collagen, but this study has shown that this is overly simplistic. Since turnover rates can vary substantially across skeletal elements and within skeletal elements throughout an individual's life, a given bone collagen sample will likely be unevenly represented over the turnover interval (Figures 6, 11, and 12). It is important to take the shifting nature of bone turnover rates into account when interpreting stable isotope data in light of diet and life history information.

Consistent with a growing body of evidence demonstrating high rates of intrabone turnover across mammalian species (Matsubayashi and Tayasu 2019; de Gruchy et al. 2024), the differences in collagen turnover rate between skeletal elements provide new research opportunities and allow for the consideration of temporal shifts in an individual's life without the reliance on incrementally forming, but inert tissues like hair, nails, and teeth. These differences are likely present across adult age ranges, though their magnitude may be amplified in older individuals, where cumulative remodeling produces more pronounced intrabone variation. Elements with faster turnover rates, such as the vertebra and pelvis, can reveal information about the period immediately before death, while bones with slow turnover rates (e.g., long bones of the arm) offer insights into a much more long‐term average. The cranium could also be a suitable bone to sample from to achieve early life signals from collagen, and the petrous portion of the temporal bone has already been suggested to reflect early life patterns of an individual (Jørkov et al. 2009). This region, if enough collagen can be extracted, could make for good samples reflecting early life, in place of long bones of the arm. Trabecular bone is also likely a good indicator of short‐term diet and can be sampled to target diet closer to death; however, comparisons of trabecular to cortical bone should be limited to those made within the same skeletal element, as the trabecular bone of one skeletal element can still have slower collagen turnover rates than the cortical bone of a different skeletal element.

Importantly, although ribs are often chosen as short‐term diet indicators and femora are commonly used to characterize long‐term diet, this study found no statistically significant difference in their turnover rates. Variations in isotopic compositions between these elements may be due to intra‐element variation, as highlighted by the multiple femur samples from the same individual. Despite similar findings of comparable turnover rates in ribs and femora (Fahy et al. 2017; Jørkov et al. 2009; Quinn 2023), the frequent use of these paired elements to show diachronic variation remains problematic and should be replaced with the ulna and vertebrae.

## 4 Conclusions

This study provides the most extensive compilation of bone collagen turnover rates across various human skeletal elements to date. With this better understanding of bone remodeling, researchers can better assess the temporal dimension of their isotopic analyses, enhancing the accuracy of their interpretations. Moreover, these findings improve the precision of diachronic analyses and offer valuable insights into factors like activity level and sampling site, which influence turnover rates. A major finding of this study was that the rib and the femur showed no significant difference in turnover rates and that if the goal is to look at diachronic change in an individual through sampling multiple skeletal elements, this approach is ill‐suited as both bones appear to have comparably intermediate turnover rates. A better approach would involve a bone with a much slower turnover rate such as the ulna or parietal and another with a much faster turnover rate such as the vertebra or pubic symphysis. In addition to considerable inter and intra‐element variation, bone collagen does not reflect the averaged diet over the period of tissue formation, as the rate of synthesis of collagen is unevenly weighted throughout life.

Future research should use a similar ^14^C‐based approach to look at a broader range of ages in humans to better understand how turnover rate changes through life. Expanding this method to better quantify the turnover rates of animal skeletal elements, especially for those with long lifespans, is also a crucially important but overlooked area of study.

## Author Contributions

**Olivia Hall:** conceptualization, investigation, writing – original draft, methodology, visualization, writing – review and editing, formal analysis, data curation. **Shari L. Forbes:** resources, writing – review and editing. **Paul Szpak:** writing – review and editing, project administration, resources, supervision, funding acquisition.

## Funding

This work was supported by Canada Research Chairs and Natural Sciences and Engineering Research Council of Canada (2020‐04740, DH‐2022‐00198).

## Conflicts of Interest

The authors declare no conflicts of interest.

## Supporting information


**Table S1:** Atmospheric 14CO2 levels for the northern and southern hemispheres. Data are from Hua et al. (2022).
**Table S2:** Calibrated isotopic and elemental data for all bone collagen samples analyzed in this study. GSN = greater sciatic notch, IC = illiac crest, IPR = ishiopubic ramus, PS = pubic symphysis.
**Table S3:** The measured Δ14C content from the skeletal elements of each REST[ES] donor, along with the estimated decay constants and the percentages of bone that was formed in each decade of life. *Z* = Decay constant, expressed as a percent. N/A indicates that the donor was not alive for the specified decade.
**Table S4:** Sample information, Δ14C values, and the estimated decay constant (*Z*) (Data from Ubelaker et al. 2022). Samples that had Δ^14^C values too high to produce a decay constant were highlighted red. *Sample had a measured Δ^14^C that was lower than any atmospheric level that existed during this individual's life. This is an impossibility and is assumed to be an error associated with sampling processing or AMS measurement.
**Table S5:** Sample information, Δ^14^C values, and the estimated decay constant (*Z*) (data from Johnstone‐Belford et al. (2022). *Individuals that were too old to produce a single reasonable decay constant do not have a decay constant indicated. Those that were older than 18 before the major spike in atmospheric levels around 1960, were excluded from this analysis due to the possibility of pre‐spike 14C remaining in the individuals.
**Table S6:** The average percent of collagen that was produced in each decade of life at death, across the elements analyzed from the southern hemisphere studies (studies Ubelaker et al. (2022) denoted with a “U”; Johnstone‐Belford et al. (2022) denoted with a “J”).
**Table S7:** Upper and lower range annual turnover rates for males and females calculated from the suggested values from Hedges et al. (2007). Inflection points are highlighted in yellow.

**Data S1:** ajpa70199‐sup‐0002‐Supinfo1.txt.

**Data S2:** ajpa70199‐sup‐0003‐Supinfo2.docx.

## Data Availability

The data that supports the findings of this study are available in the Supporting Information of this article.
