# Supplementary material for: Variation in Human Bone Collagen Turnover Among Skeletal Elements
Source: Am J Biol Anthropol. 2026 Jan 21;189(1):e70199. doi: 10.1002/ajpa.70199 (PMC12820750; doi:10.1002/ajpa.70199)
Supplement: Supplementary file 3 — Data S2: ajpa70199‐sup‐0003‐Supinfo2.docx. [file AJPA-189-e70199-s003.docx]

Supplemental study details

The samples used in these studies were from the southern hemisphere, where the radiocarbon spike in the 1950s was less pronounced compared to the northern hemisphere. The date of deaths for these individuals included 2006, 2010, and 2013-2018. The Ubelaker et al. (2022) study utilized 68 samples from six females and eleven males who were all born in 1963 and died at age 43 to 54. Four elements were sampled from each person: the parietal, the occipital, the femur, and the vertebra. The study by Johnstone-Belford et al. (2022) included 72 samples in total across 18 individuals. This study assessed differences in Δ14C for cortical and trabecular bone in the femur and rib of each individual. Of the 18 sampled individuals, 3 were female and 15 were male, and they ranged in age from 64 to 94, with the dates of death ranging from 2016 to 2018. Those that were older than 18 before the major spike in atmospheric levels around 1960, were excluded from this analysis due to the possibility of pre-spike 14C remaining in the individuals.
